# Repurposing Ketamine in Depression and Related Disorders: Can This Enigmatic Drug Achieve Success?

**DOI:** 10.3389/fnins.2021.657714

**Published:** 2021-04-30

**Authors:** Ezio Carboni, Anna R. Carta, Elena Carboni, Antonello Novelli

**Affiliations:** ^1^Department of Biomedical Sciences, University of Cagliari, Cagliari, Italy; ^2^Unit of Paediatrics, ASST Cremona Maggiore Hospital, Cremona, Italy; ^3^Department of Psychology and University Institute of Biotechnology of Asturias, University of Oviedo, Oviedo, Spain; ^4^Sanitary Institute of the Princedom of Asturias, Oviedo, Spain

**Keywords:** depression, anxiety, suicide, glutamate, esketamine, drug repositioning

## Abstract

Repurposing ketamine in the therapy of depression could well represent a breakthrough in understanding the etiology of depression. Ketamine was originally used as an anesthetic drug and later its use was extended to other therapeutic applications such as analgesia and the treatment of addiction. At the same time, the abuse of ketamine as a recreational drug has generated a concern for its psychotropic and potential long-term effects; nevertheless, its use as a fast acting antidepressant in treatment-resistant patients has boosted the interest in the mechanism of action both in psychiatry and in the wider area of neuroscience. This article provides a comprehensive overview of the actions of ketamine and intends to cover: (i) the evaluation of its clinical use in the treatment of depression and suicidal behavior; (ii) the potential use of ketamine in pediatrics; (iii) a description of its mechanism of action; (iv) the involvement of specific brain areas in producing antidepressant effects; (v) the potential interaction of ketamine with the hypothalamic-pituitary-adrenal axis; (vi) the effect of ketamine on neuronal transmission in the bed nucleus of stria terminalis and on its output; (vii) the evaluation of any gender-dependent effects of ketamine; (viii) the interaction of ketamine with the inflammatory processes involved in depression; (ix) the evaluation of the effects observed with single or repeated administration; (x) a description of any adverse or cognitive effects and its abuse potential. Finally, this review attempts to assess whether ketamine’s use in depression can improve our knowledge of the etiopathology of depression and whether its therapeutic effect can be considered an actual cure for depression rather than a therapy merely aimed to control the symptoms of depression.

## Introduction

Ketamine was originally used as an anesthetic drug in the 60s (Domino et al., 1965), but soon after, its widespread diffusion as a recreational drug posed a serious and continuing concern (Liao et al., 2017). At the beginning of this century ketamine was brought to general attention for its capacity to overcome the delay of the therapeutic action of standard antidepressants, which is one of the major problems in depression therapy (Berman et al., 2000). The fast action of ketamine has produced a vast number of reports that have tried to scrutinize several aspects of the intriguing mechanism of action of this drug on depression. Undoubtedly, the repurposing of ketamine in the therapy of depression has opened up a whole new arena in a field where the monoamine reuptake blockers have, for about 50 years, represented the go-to therapy for depression and correlated illnesses such as anxiety and post-traumatic stress disorder (PTSD).

On the other hand, the still poorly understood etiopathology of depression is reflected in the incomplete knowledge of the delayed therapeutic effect of conventional antidepressants, as well as by the poor results in a high percentage of patients, in fact, approximately only two-thirds show a marked decrease in depressive symptoms (Rush et al., 2006). Above all, the lack of an adequate animal model of depression has largely hindered the research in this field. In such a scenario, understanding ketamine’s mechanism of action has the potential to markedly improve the knowledge of the etiology of depression and may lead the way to selecting new, more efficacious and safer antidepressants (Harmer et al., 2017; Chaki, 2017). Understanding the role of reduced glutamate (NMDA) transmission in the antidepressant effects of ketamine is not an easy task because this transmission plays a key role in most brain areas and involves several other neurotransmitters (Murrough et al., 2017); this picture is complicated by the fact that also ketamine metabolites may play a role in its rapid antidepressant action (Zanos and Gould, 2018; Yang C. et al., 2018). In addition, it is necessary to consider that other pathways, whether or not directly related to NMDA receptor-mediated transmission, such as AMPA, BDNF, eEF2, glycogen synthase kinase 3 (GSK-3β), and mammalian target of rapamycin complex 1 (mTORC1), may be specifically implicated in the antidepressant actions of ketamine (Strasburger et al., 2017). Among the effects that ketamine induces, it seems possible to distinguish very early effects that may be common to the stimulating actions of ketamine, and enduring effects that may be more easily framed in its antidepressant actions. These effects may acquire relevance in depressed patients because they occur in a background of altered synaptic connectivity (Duman et al., 2019). In this review we will discuss ketamine’s mechanism of action in relation to the brain areas that may be targeted to produce antidepressant effects. We will specifically discuss the relationship between ketamine and brain circuitry involved in stress and in depression therapy, with the aim of shedding light, not only on the etiology of depression, but also on the development of potential new therapies for its treatment.

## Brief Introductory Note on Depression

Major depression is a frequent psychiatric disorder depicted as a subjective multifactorial distress; it affects overall 6% of population and has a high societal cost (Malhi and Mann, 2018). Although the etiology of depression is still undefined, the view that it may emerge from the interaction of genetic and epigenetic factors is widely accepted (Lopizzo et al., 2015). Despite this, the way this interaction could functionally affect neuronal circuitry is still debated, as is the precise role that specific neurotransmitters and mediators play in depression (Feder et al., 2009). Among epigenetic factors, juvenile traumas, adolescent stress and family frictions, and their interaction with genetic predisposition, may trigger the appearance of depression disorder (Mandelli et al., 2015). Alternatively, a genetic susceptibility can be maintained submersed by family and environmental protective conditions; likewise a genetic resilience predisposition can compensate for a genetic susceptibility (Han and Nestler, 2017; Uchida et al., 2018). Although an enduring effort has been accomplished to identify one or more specific brain areas that govern the development of depression, the complexity of its pathogenesis and the limited response to classic antidepressants have hindered the achievement of fully satisfactory results. Nevertheless, the investigations on the role of excitatory neurotransmission in brain (Thompson et al., 2015) and the rapid increase in reports evaluating the antidepressant actions of ketamine (Ionescu et al., 2021) have definitively improved the knowledge about the brain areas and circuitry involved in depression and the effects of antidepressants. In separate paragraphs, below we will discuss the role of the main brain areas involved in depression and in the actions of ketamine, the mechanism of action of ketamine and some differences between ketamine’s effects and that of classic antidepressants.

## Brief Ketamine’s History and Features

**FIGURE 1:**
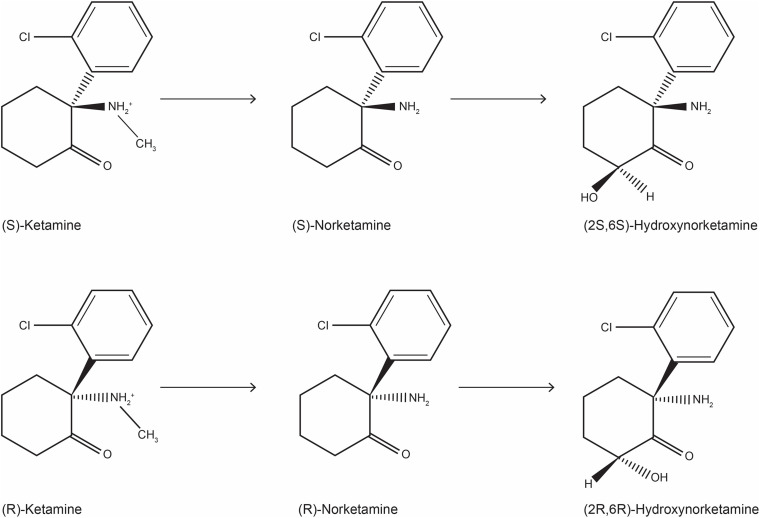
Chemical structure of ketamine stereoisomers and metabolites that can be formed following ketamine infusion in humans.

Ketamine is an arylcycloexylmine (Figure 1) that has been synthetized in the 60s as a derivative of phencyclidine (McCarthy et al., 1965); approved as an anesthetic drug in the 70s, ketamine has soon encountered a wide abuse as an illegal drug (Liao et al., 2017). Later, ketamine’s use has been extended to analgesia for acute, chronic pain and cancer pain, and to the treatment of addiction (Jonkman et al., 2017; Jones et al., 2018). The typical dissociative effect of ketamine, observed in patients and street users, encouraged scientists to explore its complex mechanism of action and its interaction with the CNS (Tyler et al., 2017). Ketamine is water-soluble anesthetic approved for specific pediatric procedures and for adult patients at risk for hypotension (Dahmani et al., 2011; Marland et al., 2013); this indication may be justified by the fact that ketamine increases the blood pressure, heart rate and cardiac output, although an action mediated by central and peripheral catecholamine reuptake inhibition is debated (Graf et al., 1995; Liebe et al., 2017; Szarmach et al., 2019). Ketamine rapidly produces a hypnotic state, profound analgesia and anesthesia, without reducing breathing act. Ketamine is a bronchodilator and is particular indicated for patients at risk for bronchospasm (Golding et al., 2016). Moreover, it produces amnesia, although the eyes may stay open, and may cause spontaneous limb movement, causing a condition defined “dissociative anesthesia” (Marland et al., 2013). Moreover, ketamine can interact with the opioid system reducing the development of tolerance induced by the long-term use of morphine (Jones et al., 2018).

The use of ketamine is associated with several side effects that make the patient evaluation unsatisfactory; among them can be observed hallucinations, intense dreams, delusions and emergence delirium that can respond to benzodiazepine treatment, (Molero et al., 2018). Overall, ketamine is considered a satisfactory anesthetic; therefore the repurposing of ketamine as a fast-acting antidepressant has been a true breakthrough (remarkably, significant antidepressants effects are observed within 24 h). However, ketamine’s approval by FDA has been the culmination of an extensive research that has investigated the role of glutamate transmission in depression pathophysiology and therapy for more than 20 years (Murrough et al., 2017). Interestingly, as early as 1999, ketamine has been shown to possess effects overlapping those of imipramine in animal model of depression (Chaturvedi et al., 1999); these antidepressant-like effects in rodents were widely reproduced later and 10 mg/kg of (R,S)-ketamine was the most frequently used dose (Polis et al., 2019). The first report demonstrating the rapid antidepressant effect of ketamine dates back in 2000 (Berman et al., 2000); in particular, these authors reported a significant improvement in depressive symptoms within 72 h after infusion of ketamine, but not placebo, in drug-free patients who had not taken any medication for at least 2 weeks. Later, the safety and the efficacy of repeated ketamine i.v. infusion (0.5 mg/kg, 40 min), were demonstrated by several studies in TRD patients (e.g., aan het Rot et al., 2009; Murrough et al., 2013). Similar results were obtained in a randomized controlled trial by administering intranasal ketamine (50 mg), (Lapidus et al., 2014). Given this unique property of ketamine, its approval has provided an alternative or a complementary treatment to classic antidepressant drugs in the therapy of depression. In fact, classic antidepressants, mostly monoamine reuptake inhibitors, are characterized by a delay of several weeks to months before clinical improvement is observed; in addition, a substantial proportion of major depression disorder (MDD) patients do not respond to reuptake inhibitors (Ferrari and Villa, 2017). Patients who do not respond to two or more antidepressant treatments are classified as treatment resistant depression (TRD), and are considered the patients of choice for ketamine’s therapy (Mrazek et al., 2014).

## Esketamine: Clinical Use in Depression

Accumulating evidence implies that N-methyl-D-aspartate (NMDA) receptor antagonism by ketamine produces rapid and sustained antidepressant activity in TRD patients (Sattar et al., 2018). Ketamine preparations (e.g., ketalar^®^, ketavet^®^) are a mixture of two enantiomers, and were approved for any use by FDA in 1970. Interestingly, the (S) enantiomer displays 4 times higher affinity for the NMDA receptor (White et al., 1985). Although most of the available data on ketamine are referred to the racemic preparations, recent preclinical and clinical studies have shown a difference in the activity of the S and R form of ketamine. Masaki et al. (2019) found that (S)- and (R)-ketamine induce completely different functional magnetic resonance imaging response pattern in conscious rats and in particular, (S)-ketamine produced a significant positive functional magnetic resonance imaging response in the cortex, nucleus accumbens (NAc), and striatum; in general (S)-ketamine parallels the effect of racemic ketamine and that of the NMDA antagonist MK-801. In addition, a recent report has shown that (S)-ketamine (15 mg/kg) produced a dose dependent activation of pyramidal medial prefrontal cortex (PFC) neurons, assayed using a genetically encoded calcium indicator (GCaMP6f) in mice (Hare et al., 2020); in particular the effect of (S)-ketamine, that has a higher affinity for the NMDA receptor channel, was similar to that produced by a 30 mg/kg dose of (R,S)-ketamine while compounds with low NMDA affinity, such as (R)-ketamine (15 mg/kg) and the ketamine metabolite (2R,6R)-HNK (30 mg/kg), had little or no GCaMP6f measured activity in pyramidal medial PFC neurons (Zanos et al., 2016). In this regard it should be mentioned that this activity is considered necessary for the rapid antidepressant response to ketamine in rodents and humans (Hare and Duman, 2020). Interestingly, several years after the first observation of the antidepressant effects of ketamine infusion (Berman et al., 2000), a pilot study by Paul et al. (2009) observed that the S-ketamine isomer did not cause the psychomimetic side effects that were observed with the racemic ketamine infusion. Later an elegant double-blind, placebo-controlled study involving 30 patients observed a rapid (within 2 h) and robust antidepressant effect of esketamine (0.2 and 0.4 mg/kg 40 min infusion), although no clear dose dependence was observed (Singh et al., 2016). Based on this evidence, (S)-ketamine, approved by FDA with the name of Esketamine, was selected for the treatment of TRD patients, (Yang et al., 2019a). The use of esketamine (commercialized in the United States with the name of Spravato), is only available within a restricted distribution system and is reserved for TRD patients. Intranasal esketamine determines a significant rapid improvement of symptoms of depression when it is given in addition to a standard antidepressant. It also reduces suicidal ideation in depressed patients at imminent risk for suicide (Canuso et al., 2018). FDA approval (Turner, 2019) recommends that patients who receive esketamine must be monitored for at least two hours, to assess eventual adverse reactions to the drug. In fact, patients may be at risk for sedation, reduced attention, dissociation (i.e., judgment and thinking alteration, depersonalization and de-realization), misuse or abuse, or even suicidal thoughts after the administration of the drug. However, it should be noted that the dissociative effects of ketamine appear with less intensity when ketamine is administered repeatedly (Singh et al., 2016). Therefore, patients should not leave the health center without the approval of the health care provider, or take the nasal spray home, nor should they drive, or operate heavy machinery for the remainder of the day of drug administration.

Overall, the rapid and sustained antidepressant effect, of ketamine (Berman et al., 2000; aan het Rot et al., 2009; Murrough et al., 2013; Lapidus et al., 2014) and esketamine (Paul et al., 2009; Singh et al., 2016) have been clearly demonstrated in drug free patients. Nevertheless, most of recent clinical trials have tested the efficacy of adjunctive intranasal esketamine in MDD or TRD patients who continued (or started) the oral antidepressant therapy (Canuso et al., 2018; Ochs-Ross et al., 2020; Citrome et al., 2020; Singh et al., 2020). In particular the efficacy of intranasal esketamine was evaluated in three short term (four week) clinical trials and one long-term maintenance-of-effect trial, in which patients received a new oral antidepressant that was continued throughout the trial (Daly et al., 2018). In one short-term trial, esketamine reduced significantly the severity of depressive symptoms. In the long-term trial, patients in stable remission upon continuation of esketamine in association with an oral antidepressant experienced a statistically significant longer interval without depressive symptoms, when compared with patients who received the placebo nasal spray plus the oral conventional antidepressant (Daly et al., 2018). In summary, as reported by a recent meta-analysis, of placebo controlled trials in MDD/TRD patients, intranasal esketamine determined a rapid antidepressant effect and was relatively safe and tolerable, although the long-term therapeutic effect and safety need further confirmation (Wei et al., 2020).

On the other hand, evidence of an antidepressant action of (R)-ketamine and (2R,6R)-HNK (hydroxynorketamine) has been provided; in particular (R)-ketamine, although four-fold less potent as NMDAR antagonist than (S)-ketamine, has shown more marked and longer-lasting antidepressant-like effects than the (S)-enantiomer in several animal models of depression (Yang et al., 2019a). In addition, preclinical studies assessing locomotor activity, prepulse inhibition and conditioned place preference, have suggested that (R)-ketamine would be a safer antidepressant than (R,S)- or (S)-ketamine (Chang et al., 2019). The mechanisms involved in the antidepressant action of (R)-ketamine have been reviewed recently (Hashimoto, 2020; Jelen et al., 2021); these authors outlined, among others, the role of transforming growth factor β (TGF-β), ERK activation, tropomyosin kinase B signaling, mTORC1, the beneficial role on alterations in the gut microbiota, and spleen, concluding that (R)-ketamine has fewer harmful side effects than (R,S)-ketamine or (S)-ketamine in rodents, monkeys, and humans. Interestingly, Leal et al. (2020), in a pilot study in TRD patients observed that (R)-ketamine (arketamine) might produce a fast (60 min) and sustained antidepressant effect (7 days). On the other hand, Passie et al. (2021) in an interesting placebo-controlled study in healthy volunteers (*n* = 10), found no significant difference between the neuropsychological and psychopathological effects of two equivalent doses of (R,S)-ketamine and (S)-ketamine. Therefore, on the basis of this consideration it is likely that the choice of the right ketamine’s enantiomer for the treatment of TRD or other forms of depression will be further debated (Hashimoto, 2019).

## Ketamine and Suicidal Behavior

Suicide is a relevant cause of death among people worldwide, in particular amid those suffering of psychiatric disorders. Suicidal behavior is a composite and multifactorial occurrence, mostly generated by extreme distress (Nugent et al., 2019); it can be triggered by a sudden event, not linked to a psychiatric pathology (e.g., economic desperation) or can be the result of an escalating behavior that occurs within a mental illness, such as depression or schizophrenia (Bachmann, 2018).

A distinctive feature of MDD is the frequent manifestation of suicidal thoughts and attempts; it can be quantified in a suicide risk of 55%, although other concomitant psychiatric disorders, such as borderline and antisocial personality and violent behavior can contribute to the appearance of suicidal behavior (Olfson et al., 2017). Unfortunately, no validated biochemical markers can predict suicide and although a failed attempt is a strong suggestion of a further attempt, few behavioral signs can be truly predictive as they often overlap with depression symptoms (Sudol and Mann, 2017). Yet, a systematic review (Miná et al., 2015) reported that inflammatory cytokines can be useful markers of suicidal ideation; in particular IL-6 appears to be elevated in the cerebrospinal fluid of suicide attempters and even peripheral blood levels have been proposed as a biological suicide marker. On the other hand, the only FDA approved drug for suicide prevention is clozapine, an antipsychotic drug used in the therapy of schizophrenia (Vermeulen et al., 2019), although lithium also has a clear preventive effect when used in mood disorders (Cipriani et al., 2013). This evidence highlight two main gaps: (i) a better understanding of the brain circuitry responsible for the formation of suicidal ideas and the execution of suicidal acts is needed; ii) there is an enormous need of new drugs to be used for controlling or preventing suicidal ideation. In particular the distinction of risk factors associated with suicidal ideation or suicidal attempts would be a useful tool in preventing suicidal attempts (Klonsky et al., 2018).

In this context, the idea of using ketamine to treat the disorders that underlie suicidal ideation has been shown to be more than a working hypothesis and could represent a real preventive therapy. In fact, although ketamine’s mechanism of action has not been fully clarified, there is no doubt that ketamine is effective in reducing suicidal ideation and attempts. Several studies demonstrated that either 0.5 mg/kg of ketamine infused over 40 min versus placebo (Zarate et al., 2012a; Burger et al., 2016; Hu et al., 2016) or versus midazolam (Price et al., 2014; Murrough et al., 2015; Fan et al., 2017; Grunebaum et al., 2018, 2017), definitively reduced suicidal ideation and depressive symptoms. A recent report showed that also intranasal esketamine (84 mg twice a week for 4 weeks), in addition to comprehensive standard of care treatment, may result in a significant reduction of depressive symptoms and suicidal ideation (Canuso et al., 2018). In this regard, it is intriguing to consider the role of nightmares in the prediction of suicidal behaviors; in fact, nightmares have been associated with suicidal behavior separately from concomitant psychiatric diseases such as depression, anxiety and PTSD (Titus et al., 2018). Therefore, it is of great interest understanding whether the dissociative effect of ketamine triggers some mechanism that interfere with the generation of nightmares in the CNS, or interferes in the transition process between the ideation of suicide and its realization; the understanding of these mechanisms would be of great help in the search of a therapy to prevent suicide. In this regard, Vande Voort et al. (2017) reported that among patients who received a single ketamine infusion (0.5 mg/kg over 40 min), those who showed an anti-suicidal response had significantly reduced nocturnal wakefulness the night after ketamine infusion, when compared with those not showing an anti-suicidal response. Considering that the effect of ketamine on suicidal ideation is intimately related to the antidepressant effect, it is stimulating to think that the effect of ketamine on nightmares and on the reduction of wakefulness may be somehow related to the dissociative effects of ketamine. In fact it has been reported that dissociation could predict a robust and sustained antidepressant effect (Luckenbaugh et al., 2014; Niciu et al., 2018). However, one must consider that the latter correlation is debated (Ballard and Zarate, 2020) and that dissociative effects and sleep effects occur at different times (Vande Voort et al., 2017). Furthermore it is possible that ketamine may produce different effects in depressed and suicidal patients than in individuals without these conditions; in fact, ketamine can cause unpleasant dreams in healthy volunteers over the three post-administration nights (Blagrove et al., 2009) and has not decreased delirium in older adults undergoing negative experiences after major surgery (Avidan et al., 2017).

In general, and probably because of the extent of glutamate transmission in the brain, it can be suggested that the response to ketamine is closely related to the emotional state of the individual and therefore both rapid and prolonged effects can be very different between individuals; in this regard, Aust et al. (2019) with an interesting study, have shown that the state of anxiety induced by ketamine in depressed patients is predictive (inversely proportional) of the antidepressant response of ketamine. In addition, the different individual response could still be different following the administration of (R-S)-, (S)-, or (R)-ketamine administration (Passie et al., 2021). In contrast with the two most used drugs for preventing suicidal behavior (i.e., clozapine and lithium), which require weeks for producing their beneficial effect, ketamine can reduce suicidal ideation rapidly (Vermeulen et al., 2019). Moreover, although ketamine shows a common antidepressant effect with the monoamine reuptake blockers, these drugs reduce risk of suicidal ideation in old but not in young patients (Carpenter et al., 2011); this suggests that ketamine’s effect on suicidal behavior may involve peculiar brain mechanisms. Regrettably, despite the great interest in the fast-antidepressant and anti-suicidality effect of low ketamine doses, very little is known on the brain circuitry involved in ketamine’s effect. Several interesting reports highlighted the involvement of opioid receptors. In particular it was shown that naltrexone reduces the antidepressant effect of ketamine (Williams et al., 2018) as well as the anti-suicidality effect (Williams et al., 2019). Although these studies did not differentiate among opioid receptors MOR, DOR or KOR, in the light of the anti-suicidality effect of buprenorphine, the authors speculated that a partial or a brief period of activation of the opioid system by ketamine is associated with an anti-suicidality effect, whereas full and chronic opioid system activation is associated with an increase in suicidality.

One interesting aspect of suicidal behavior is the passage between the ideation to execution; the last requires the involvement of the decision-making brain area and the activation of the related neurotransmitter release. Interestingly, suicide is more prevalent among men, whereas non-fatal suicidal attempts are more prevalent among women, young people, and generally among individuals unmarried or bearing psychiatric disorders (Nock et al., 2008). Consistent with this evidence, assessment of decision-making in preclinical and clinical studies revealed a difference between genders; in particular, female rats perform better and improve less during sessions than males, when tested through the Iowa gambling task (Georgiou et al., 2018). In addition, this study demonstrated the relevance of dopaminergic pathways in the gender difference and suggested that female are less able to cope with stress during the test, leading to maladaptive decision-making, (Georgiou et al., 2018). It might be generally summarized that women under stress have their decision making more damaged, and thus may be more prone to act for committing suicide under stress, but interestingly, at the same time, are more responsive to the pharmacological antagonism of CRF-induced activation of the hypothalamic-pituitary-adrenal axis (HPA) (Webster et al., 1996) or to anxiolytic drug (Zorrilla et al., 2002). On the other hand, stress negatively affects decision making differently for men and women; men’s performance in the IOWA test deteriorates as stress levels increase, whereas the performance of women improved to a point and then deteriorates as stress levels increase (van den Bos et al., 2009; Wemm and Wulfert, 2017). In addition decision-making has been found altered in suicide attempters (Jollant et al., 2005, 2007). Overall, considering that according the WHO, in 2015 800,000 suicides occurred worldwide, and that suicides account for 1.4% of premature deaths worldwide (Bachmann, 2018), the proved efficacy of ketamine in reducing suicidal behavior is a breakthrough in managing this worldwide public health concern.

## Current Use and Potential for Ketamine Use in Resistant Depression in Adolescence and Childhood

Depression and anxiety are common conditions in childhood and adolescence; globally the prevalence of these two disorders in the 5–17 age group is 6.16% and 3.2% respectively (Erskine et al., 2017). Additionally, the suicidal rate among adolescent aged 12–17 years was 5.2/100 000 in 2014 (Sheftall et al., 2016) and suicide represents the second leading cause of death in the United States among individuals aged 10–24 (Kim et al., 2020). Pediatrics depression is also associated with poor academic performance, social disease, early pregnancy, physical illness and substance abuse (Fergusson and Woodward, 2002; Keenan-Miller et al., 2007). When depression is diagnosed in elementary school, the therapeutic approach is a complex problem to deal with, as it is not possible to predict the evolution of the disease and the influence of environmental conditions that can change its course. It has been recently suggested that preschool depression was a highly salient predictor of prepubertal and mid-to-post pubertal MDD (Gaffrey et al., 2018); these authors, in an elegant study observed that children with a history of preschool depression continued to show clear depressive symptoms from childhood to adolescence, highlighting the clinical significance and public health outcome of an early diagnosis and cure of depression at childhood and prepubertal age. Generally, the efficacy of antidepressant therapy in children and adolescents has been established (Walkup, 2017); the TADS (Treatment for Adolescents with Depression Study) study revealed that over a 6 – 9 months treatment period [fluoxetine, cognitive behavior therapy (CBT) or their combination], 80% of participants experienced symptom improvement (March et al., 2007) while the TORDIA (Treatment of Resistant Depression in Adolescents) study showed that more than 60% of participants remitted when selective serotonin reuptake inhibitors (SSRI), venlafaxine, CBT or their combination were administered (Vitiello et al., 2011). Hereafter, the first line for moderate to severe youth depression recommends a multimodal approach that consists of a combination of psychotherapy and pharmacotherapy [i.e., selective serotonin reuptake inhibitors (SSRI)], (Clark et al., 2012). Although SSRI are clearly efficacious, in the case of non-responders, a switch to an antidepressant with a different mechanism (e.g., venlafaxine) and a cognitive behavioural therapy (CBT) resulted in a higher rate of clinical response than switching to another medication without CBT (Brent et al., 2008). Nonetheless, a consistent number of adolescent patients that do not respond even after two medications treatments with CBT, can be categorized as TRD; they require diagnostic careful evaluation, clinical attention and innovative therapies (Dwyer et al., 2020). Among new therapies, several studies investigated the efficacy of ketamine in children and adolescent with TRD (Papolos et al., 2013; Dwyer et al., 2017; Cullen et al., 2018; Zarrinnegar et al., 2019). In particular Cullen et al. (2018) administered ketamine (0.5 mg/kg; six i.v. infusion in 2 weeks) to 13 TRD adolescents aged 12–18; overall they observed that 5 subjects responded and remained in remission at a 6 week check-up, while 2 were still in remission after 6 months. Cullen et al. (2018), further reported that dissociative symptoms vanished within 1 h after ketamine administration and that the drug was generally well tolerated. In a retrospective study Papolos et al. (2013) observed that intranasal ketamine administration in 12 treatment resistant youths (ages 6–19 years) with bipolar disease-fear of harm (a pediatric onset phenotype o bipolar disease, BD-FOH) was well tolerated and produced a significant improvement in mood, anxiety, and behavioral symptoms such as mania and aggression. In an another report, Papolos et al. (2018) observed that the administration of intranasal ketamine in 45 youths with refractory BD-FOH was efficacious and well tolerated supporting the potential effectiveness of ketamine therapy; nevertheless these authors underlined that the date presented were preliminary, neither blind nor placebo-controlled, therefore must be interpreted with caution (Papolos et al., 2018). An interesting case-report on the use of repeated ketamine i.v., in a 16-year old male with psychiatry history of resistant major depressive disorder (MDD), has been reported by Dwyer et al. (2017); the patient experienced an immediate reduction (within 1 day) of depression symptoms, suicidal ideation and hopelessness, that lasted for the hospitalization period (8 weeks), which allowed the discharge of the patient, after psychiatric stabilization, with a plan to receive further ketamine infusion every 3–6 weeks, along with a pharmacotherapy and psychotherapy support. Repeated ketamine i.v. infusion was also proved to be efficacious in a 15-year-old adolescent female with TRD, generalized anxiety disorder and PTSD secondary to sexual trauma (Zarrinnegar et al., 2019); even in this case, ketamine (0.5 mg/kg; six i.v. infusion in 2 weeks) reduced significantly depressive symptoms, and suicidal ideation and the girl could be dismissed with a pharmacotherapy support because a resolution of depressive and psychotic symptoms was achieved and maintained for several months.

A systematic review of these and other studies was elegantly provided by Kim et al. (2020); these authors suggested that ketamine generally shows the potential to be effective in reducing depressive symptoms, acute suicidal behavior and mood lability in the youth with TRD and bipolar disease, being well tolerated in the pediatric cohort with minimal side effects. However, these authors also acknowledged that a number of subjects did not respond to ketamine administration and highlighted that the American Academy of Child and Adolescent Psychiatry (AACAP) does not officially endorse the utilization of Esketamine for youth TRD (Kim et al., 2020). In conclusion, the extension of ketamine administration to youth TRD is needed and desirable, but it needs further and urgent evaluation; in particular the potential effect of this drug on the developing brain of children or adolescents must be carefully evaluated. In fact, the possible reduction of excitatory input on the parvalbumin interneurons of the PFC may lead to impaired cortical function (Thomases et al., 2013) in adulthood, with the potential risk of long-term adverse cognitive and emotional changes (Zimmermann et al., 2020). Of note, broadening the regimen of ketamine administration after discharge, from two or three times a week to only once every 3–6 weeks, drastically lowers the risk of long-term cognitive effects. From another point of view, in the cost-benefit assessment, we have to take into account the hypothetical damage, that symptoms of anxiety, depression, or nightmares due to a PTSD, or even suicidal thoughts or attempts, can cause to the process of brain maturation and personality formation that occurs in adolescence and pre-adult age.

## Mechanism of Action and Metabolism of Ketamine

### Interaction With Glutamate Receptors Interaction

Since 1994 it is known that chronic treatment with antidepressants or electroshock resulted in an adaptive response of cortical glutamate NMDA receptors (Paul et al., 1993, 1994). Although this evidence led to targeting glutamate signaling for developing new antidepressants (Murrough et al., 2017) and in particular to ketamine use (Berman et al., 2000), almost 30 years have not been enough to fully unveil the mechanism of action of ketamine in producing the rapid and sustained antidepressant effects. The yet inadequate knowledge of depression etiopathology, and the wide distribution of glutamatergic innervation in mammalian brain, greatly complicates the identification of a preferential site of action for ketamine, both in terms of brain area and cell type. Moreover, the variable interaction of the two enantiomers of ketamine and respective metabolites with glutamate receptors, i.e., NMDA and AMPA, and the relative receptor affinity, adds complexity to the comprehension of the mechanism of action.

The metabolism of ketamine is complex and involves an initial N-demethylation by liver microsomal cytochrome P450 into norketamine (Figure 1), with CYP3A4 being the principal metabolizing enzyme (Hijazi and Boulieu, 2002); subsequently, norketamine is further metabolized to the hydroxynorketamine (HNK) and dehydronorketamine (DHNK) (Zanos et al., 2018). Among the various stereoisomers that can be formed following ketamine infusion in human, the predominant species in plasma are (2R,6R;2S,6S)-HNK and (2S,6R;2R,6S)-HNK (Moaddel et al., 2010). Of note, norketamine, DHNK, and (2R,6R;2S,6S)-HNK were detected as early as 40 min after 0.5 mg/kg i.v. ketamine infusion for 40 min. Moreover, different concentrations of metabolites have been described in MDD or bipolar depression (Zarate et al., 2012b). Although ketamine metabolism does not occur in the brain, both ketamine and its metabolites easily pass the blood-brain barrier via a non-enantiomer-selective transport (Zanos et al., 2018).

Ketamine is a non-competitive antagonist of the NMDA receptor (Martin and Lodge, 1985; Zorumski et al., 2016), which binds to the phencyclidine site within the channel in the open state, preventing ion flow (Pham and Gardier, 2019). Among (S) and (R) enantiomers, (S) ketamine displays four-fold higher affinity for the NMDA receptor (White et al., 1985). Therefore, the administration of the (S) enantiomer is expected to produce less adverse effects than the racemic mixture (Muller et al., 2016). In addition to NMDA antagonism, Zanos et al. (2016) found that the metabolite (2R,6R)-HNK exerts behavioral, electroencephalographic, electrophysiological and cellular antidepressant-related actions in mice, via the activation of AMPA receptor and independently from NMDA receptor, which may account for the lack of ketamine-associated side effects observed with this metabolite (Zanos et al., 2016).

**FIGURE 2:**
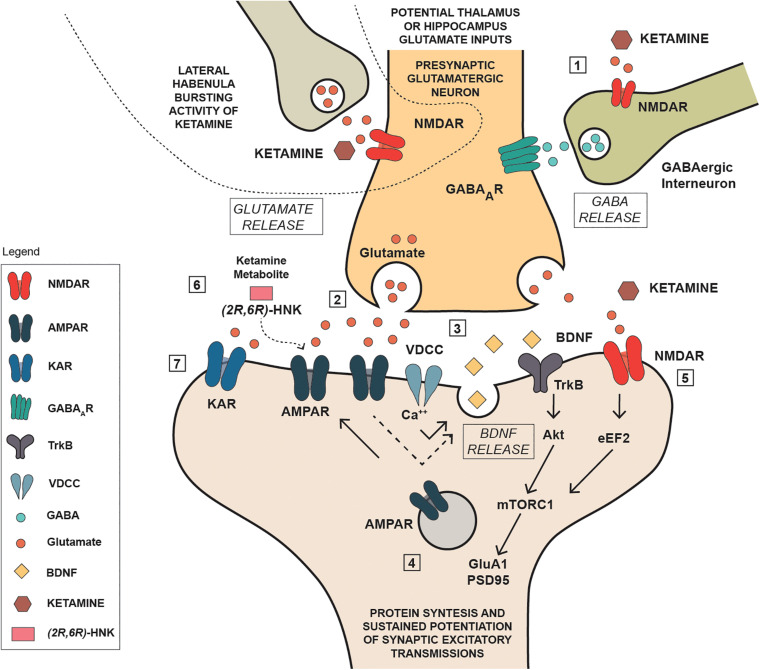
Schematic representation of some potential mechanisms involved in the antidepressant actions of ketamine. (1): Ketamine reduces NMDA receptor mediated stimulation of GABA interneurons reducing the inhibitory action on presynaptic glutamatergic neuron; (2) the reduced inhibition produces a rapid glutamate burst acting on AMPA receptors located on pyramidal neurons; (3) Such activation leads to the opening of voltage-dependent calcium channels (VDCC) that stimulate BDNF; (4) BDNF induce the translation and synthesis of key synaptic proteins in synaptogenesis and maturation of dendritic spines, including GluA1 and PSD95), via the TrkB/Flk-1 - mTORC1-signaling pathway; (5) ketamine acting as NMDA inhibited the eukaryotic elongation factor2 (eEF2) kinase, resulting in reduced eEF2 phosphorylation and increased BDNF translation and protein synthesis; (6) ketamine metabolite can stimulate AMPA receptors independently of NMDA receptor blockade by ketamine; (7) kainate receptor can contribute to Na^+^ and Ca^++^ entry, neuronal depolarization and postsynaptic responses.

Established that ketamine and its metabolites can interact with glutamate transmission, either directly through NMDA receptor blockade or indirectly through an enhancement of glutamate transmission at AMPA receptors, it becomes challenging to evaluate the consequences of such interaction and where in the brain it may occur. It is noteworthy that ketamine’s antidepressant action is generally associated with a significant increase of brain derived neurotrophic factor (BDNF), and ketamine activity on glutamate transmission may be instrumental to this effect. In a mouse model of depression, Autry et al. (2011) found that ketamine and other NMDA antagonists inducing a fast antidepressant-like behavioral effect, inhibited the eukaryotic elongation factor2 (eEF2) kinase, resulting in reduced eEF2 phosphorylation and increased BDNF translation. On the other hand, the increase in BDNF expression in the hippocampus is a typical feature of standard antidepressants (Harmer et al., 2017; Monteggia et al., 2004; Kraus et al., 2017; Björkholm and Monteggia, 2016), while it is widely acknowledged that chronic stress causes a down-regulation of BDNF protein and mRNA in the hippocampus (Zaletel et al., 2017), an effect that is strictly linked with depression (Duman and Monteggia, 2006). Interestingly, postmortem studies have shown that depressed patients have a lower brain volume and neuron density in the dorsolateral PFC (Rajkowska et al., 1999; Drevets, 2000), and a lower expression of synaptic-function related genes (Kang et al., 2012). On this basis it is reasonable that ketamine, via a glutamate-mediated effect, may improve the synaptic connectivity and trigger the functional recovery of damaged neuronal network, which is typical of depression (Duman et al., 2019; Deyama and Duman, 2020). Notwithstanding, the underlying mechanism is puzzling given the overall ketamine effect in reducing, rather than activating glutamate transmission. To this regard, a current view proposes that the initial blockade of presynaptic NMDA receptors located on GABA interneuron terminals in the medial PFC is of pivotal importance (Figure 2). GABA interneurons innervate the glutamate terminals of this region, therefore their inhibition via NMDA blockade would result in a reduced GABA release, and a consequent rapid glutamate burst acting on AMPA receptors located on pyramidal neurons that project to subcortical areas, or on other pyramidal cells (Lett et al., 2014; Deyama and Duman, 2020). Such activation leads to the opening of voltage-dependent calcium channels (VDCC) that stimulate BDNF and vascular endothelial growth factor release. BDNF and vascular endothelial growth factor induce the translation and synthesis of key synaptic proteins in synaptogenesis and maturation of dendritic spines, including GluA1 and postsynaptic density protein 95 (PSD95), via the TrkB/Flk-1 - mTORC1-signaling pathway (Duman and Aghajanian, 2012). In agreement with this evidence, Deyama and Kaneda (2020) reported that a single BDNF infusion in the medial PFC produced antidepressant-like effects that lasted up to 8 days, with an outcome very similar to the rapid and sustained antidepressant effect produced by ketamine (Björkholm and Monteggia, 2016). Adding complexity to the issue, Pham and Gardier (2019) recently suggested that ketamine may block postsynaptic NMDA receptors in the hippocampus, leading to increased BDNF production with a mechanism involving eEF2, similar to that proposed by Autry et al. (2011). They also suggested that ketamine metabolite HNK may play antidepressant activity via a direct activation of post-synaptic AMPA receptors, leading to increased extracellular glutamate and GABA levels in the medial PFC (Pham and Gardier, 2019). While this explanation is linear and satisfactory, other brain regions enriched on NMDA and AMPA receptors are likely involved in the actions of ketamine and its metabolites, given the complexity and wideness of brain glutamate innervation.

### Pharmacokinetic Considerations

An important aspect of ketamine’s mechanism of action is its ability to induce an antidepressant effect whose duration extends beyond the presence of a relevant plasma concentration (Berman et al., 2000). Therefore, it is of interest to evaluate the temporal correlation between the onset of behavioral effects of ketamine with plasma or brain concentration and the corresponding temporal variation in NMDA receptor occupancy after administration in different animal species (Shaffer et al., 2014). However, this correlation is complex because most of the preclinical and clinical experimental observations have been obtained by administering racemic ketamine, which is composed of two enantiomers that have different effects and give rise to a total of four different metabolites (see section “Interaction With Glutamate Receptors Interaction”). The single infusion of 0.5 mg/kg of (R,S)-ketamine can determine a very rapid appearance of dissociative and psychotomimetic effects that gradually disappear in 60–120 min (Krystal et al., 1994). The time interval required for the psychotomimetic effects of ketamine to appear, allows some consideration to be made.

From a pharmacokinetic and pharmacodynamic perspective, the appearance of acute psychotic symptoms, including visual and auditory hallucinations, thought disorders and apathy, within 5 min from starting S-ketamine infusion, with a reported serum concentration of this drug of 2.26 μM (539 ng/ml) (Vollenweider et al., 1997) could be attributed to the occupation > 60% of the NMDA receptor by S-ketamine. According to the elegant study by Shaffer and coll. on ketamine receptor occupancy normalization (Shaffer et al., 2014), we should consider: (1) a similar free plasma unbound concentration of (±) ketamine in rats and humans; (2) ketamine rapid and high penetration in the brain; (3) ≈60% receptor occupancy reported by Shaffer and coll. for a similar concentration of (±) ketamine in rats; (4) a 40% higher concentration of S-ketamine (Vollenweider et al., 1997) with respect to its content in the racemic mixture utilized in the receptor occupancy normalization study. This latter point is particularly important when considering the higher affinity for NMDA receptors reported for S-ketamine (Ki ≤ 0.7 μM) vs R-ketamine (Ki ≤ 2.6 μM) (Hashimoto, 2019), and the reported absence of significant psychotic symptoms following the injection of the same dose of R-ketamine (Vollenweider et al., 1997). According to the receptor occupancy normalization study, a plasma concentration of 204–229 ng/ml (0.86 – 0.96 μM) (±) ketamine generates a 31–33% receptor occupancy both in healthy volunteers and MDD patients, and such concentrations of (±) ketamine have been reported to be associated with transient psychotomimetic and dissociative symptoms, resolved within 2 h, without delusions or hallucinations (Singh et al., 2016).

As previously reported, the metabolite of ketamine (2R,6R)-HNK may contribute to neuronal firing stimulation by activating AMPA receptors (Zanos et al., 2016). Although the authors claim that the metabolite is “necessary and sufficient” to produces ketamine’s antidepressant actions, we suggest that the contribution of ketamine acting at the NMDA receptor may be more relevant than they claim. In fact, the reported concentration of brain ketamine is between two and three times that of the metabolite (2R,6R)-HNK acting as a possible AMPA agonist and the density of AMPA and NMDA receptors varies in the different regions of the brain (Can et al., 2016; Scheefhals and MacGillavry, 2018). Moreover, evidence of a AMPA receptor-activation independent role for S-norketamine has been published (Yang C. et al., 2018). Overall, pharmacokinetic and mechanistic considerations together with the likely change of receptor expression upon repeated drug treatment indicate that unveiling the relative contribution of glutamate receptors in the ketamine’s effects is a puzzling question. The BDNF-mediated increase in AMPA receptors, occurring in prolonged ketamine treatment, may strengthen the action of ketamine on these receptors, creating the conditions for the sustained effect of ketamine. While the murine model of depression does not replicate the complexity of TRD in humans, the results of an ongoing clinical trial, investigating the effect of the non-competitive AMPA receptor antagonist perampanel in TRD patients treated with ketamine, might greatly improve the understanding of this issue (ClinicalTrials.gov Identifier: NCT03973268).

## Brain Areas and Neuronal Circuitry Involved in Ketamine’s Effects

**FIGURE 3:**
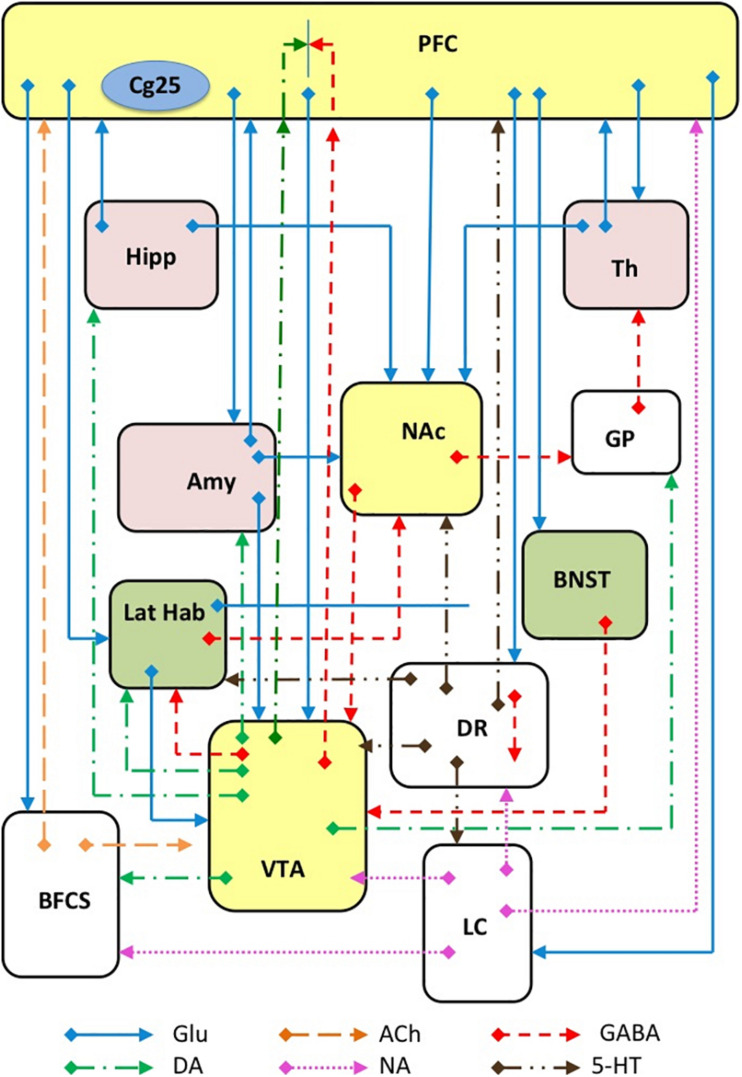
Schematic representation of several major glutamatergic input pathways on mammalian brain nuclei where ketamine might interact with glutamate transmission by interacting with NMDA and/or AMPA receptors. Abbreviation: PFC, prefrontal cortex; Cg25, subgenual cingulate region; Th, thalamus; Hipp, hippocampus; LHb, lateral habenula; Amy, amygdala; VTA, ventral tegmental area; DR, dorsal raphe; BFCS, basal forebrain cholinergic system; LC, locus coeruleus; GP, glubus pallidus. Glutamate (Glu), dopamine (DA), GABA, acetylcholine (Ach), norepinephrine (NA) and serotonin (5-HT) neurons and axons are represented.

Depression is now acknowledged as a complex disorder characterized by the involvement of many brain areas, neuronal circuits (Figure 3), neurotransmitters and intracellular mechanisms. Although it was reductive to think that the alteration of a single area could be sufficient for the generation of such a complex disorder, the search for such an area has represented a challenge for many researchers, whose ultimate goal was to better understand the etiopathogenesis of depression and thus improve the chances of developing new effective antidepressants. Moreover, the identification of a such an area would have favored the development and characterization of animal models of depression to test antidepressant drugs by ascertaining the reversibility of the changes observed. Among the areas that have been associated with depression, the hippocampus and the prefrontal cortex certainly stand out, but recently the NAc has also been involved in consideration of its role in anhedonia (inability to feel pleasure), (Keedwell et al., 2005). In particular, the study of neurogenesis in the hippocampus has been used to correlate the effects of drugs on growth factors (e.g., BDNF) with their antidepressant potential (Masi and Brovedani, 2011). The effect of ketamine in the hippocampus, PFC, NAc, and lateral habenula (LHb) will be discussed below in order to recognize changes that might be related to its antidepressant effect.

### Hippocampus

The reduction of hippocampus volume was identified as a sign of major depression and since the first report (Magariños and McEwen, 1995a, b), most studies have confirmed this observation, although few negative reports raised some controversy (Sheline et al., 2019). Interestingly, ketamine exerts antidepressant effects in TRD patients who have a relatively smaller hippocampus (Abdallah et al., 2015). The decline of hippocampal neurogenesis, a process by which normally, 700 new born hippocampal granule cells are added daily (Spalding et al., 2013), has been considered the second important biomarker of major depression, although some limits should be pondered (Miller and Hen, 2015). It has been repeatedly reported that chronic stress decreases hippocampal neurogenesis, while classic antidepressants can reverse this decrease (Czéh et al., 2002; Pham et al., 2003; Alonso et al., 2004). In addition, the reduction of neurogenesis can result in neuronal atrophy, as observed by neuroimaging and post-mortem studies in depressed patients (Bremner et al., 2000; Frodl et al., 2007; Huang et al., 2013; Wise et al., 2017).

The delay in the appearance of the antidepressant effect, after starting the therapy with standard antidepressants, has often been attributed to the time needed to fully stimulate hippocampal neurogenesis. It is therefore interesting to discuss the association between the antidepressant effects of ketamine and its neurotrophic actions. This issue has been very recently reviewed by Deyama and Duman (2020); these authors suggested that the neurogenesis might be involved in the sustained but not in the fast actions of ketamine, though further studies are required to fully clarify the effect of ketamine on hippocampal neurogenesis. Interestingly, Yamada and Jinno (2019) reported that ketamine, two days after the administration of single dose, elevated the density of neuronal progenitors and new-born granule cells in the ventral but not in the dorsal hippocampus in adult mice, although the densities of neuronal stem cells were not affected by ketamine in both areas. The evidence that ketamine affects neurogenesis only in the ventral hippocampus (vHipp) appears highly relevant because of its link with emotional processes, while the dorsal part is related to spatial memory (Bannerman et al., 2014). Noteworthy, ketamine may have a prophylactic efficacy against stress-induced depressive-like behavior, by altering the hippocampal neural activity (Mastrodonato et al., 2018). This study showed that a single injection of ketamine, administered to mice before the exposure to social defeat stress, selectively increased Δ-FosB expression in the vHipp (Mastrodonato et al., 2018).

### Prefrontal Cortex

A reduced volume of several PFC subregions has been observed in patients suffering major depression and/or exhibiting suicidal thoughts and behaviors (Schmaal et al., 2020). The ventral PFC system may be important in reducing the positive while amplifying the negative internal states that can induce suicidal ideation, while the dorsal PFC and the inferior frontal gyrus may facilitate suicidal attempts. Ketamine displays a rapid effect on neuronal plasticity in the PFC (for a detailed review see Deyama and Duman, 2020). In particular, Li et al. (2010) reported that a single low dose of ketamine rapidly activated the mammalian target of rapamycin (mTOR) signaling, resulting in sustained elevation of synapse-associated proteins and spine number in the PFC, an effect, that is the opposite of that caused by the exposure to stress. A confirmation of the involvement of PFC in ketamine’s effect has been recently provided in mice models of chronic stress and chronic corticosterone exposure (Moda-Sava et al., 2019). These authors demonstrated that a depression-related behavior was associated with branch-specific elimination of postsynaptic dendritic spines on PFC projection neurons, and that antidepressant doses of ketamine reversed these effects by selectively rescuing spines. In addition, Moda-Sava et al. (2019) highlighted that corticosterone and ketamine are both able to regulate spine remodeling at the molecular level, directing spine formation and removal through both transcription-dependent processes and rapid non-genomic-mechanisms. They also suggested that synaptogenesis could be required for the enduring antidepressant effects of ketamine, but not for rapid effects (Moda-Sava et al., 2019). These data are somehow in contrast with the report by Li et al. (2010), who observed increased levels of synaptic proteins (i.e., PSD-95, synapsin-1, and GluA1) as early as 2 h after ketamine administration. Overall, these data indicate that medial PFC pyramidal neurons and the local activity-dependent synaptogenesis play a crucial role in the antidepressant actions of ketamine (Deyama and Duman, 2020). Interestingly, Carreno et al. (2016) investigated the role of the vHipp – medial PFC pathway in the antidepressant response to ketamine, as evaluated by the forced swim test in Sprague-Dawley rats. This study observed that inactivation of the vHipp with lidocaine prevented the sustained, but not the acute antidepressant effect of ketamine, and suggested that the activity in the vHipp-medial PFC pathway is both necessary and sufficient for the antidepressant-like effect of ketamine (Carreno et al., 2016). Interestingly, Chen et al. (2019), through the assessment of the resting functional connectivity magnetic resonance imaging (fcMRI) in TRD patients, observed that ketamine (0.5 and 0.2 mg/kg) could modify fcMRI in different regions of the PFC and in particular, the increase of the functional connectivity between the right dorsolateral PFC and the left superior parietal region was correlated with the reduction of suicidal ideation in the low-dose group. Resting fcMRI was also evaluated in a study by Gärtner et al. (2019); the authors observed that a single dose of ketamine (0.5 mg/kg), administered to depressed patients, increased resting fcMRI between right PFC and sgACC and this increase is positively related to treatment response while a low baseline of functional connectivity between these regions predicts treatment outcome. In addition, recently Abdallah et al. (2018), observed that ketamine increased glutamate-glutamine cycling in the PFC of MDD patients that was correlated with a rapid antidepressant effect, an effect that could be representative of an increased neurotransmission strength in the PFC.

### Nucleus Accumbens

The NAc has been implicated in the etiology of depression because of its role in brain’s reward circuits, anhedonia and aberrant reward-associated perception (Russo and Nestler, 2013). The glutamatergic afferences that NAc receives from medial PFC, vHipp and basolateral amygdala support its crucial role in regulating the individual susceptibility to depression, and point to NAc as a target area for the antidepressant effects of ketamine. In addition, the NAc receives a GABAergic innervation from the lateral habenula and a dopaminergic innervation from the ventral tegmental area (VTA) (Russo and Nestler, 2013; Bagot et al., 2015). In particular, the central dopaminergic circuitry is acknowledged, as a crucial station for motivated behavior, hedonic appraisal and mood regulation; therefore, its potential involvement in the antidepressants effect of ketamine is more than likely. Witkin et al. (2016) have recently shown that ketamine and LY341495 [a potent and selective antagonist of group II metabotropic glutamate receptor (Kingston et al., 1998)] but not the selective serotonin reuptake inhibitor (SSRI) citalopram, increased the number of spontaneously active dopamine neurons in the VTA, and the extracellular dopamine levels in the NAc and in the PFC. These authors have shown that the effects of ketamine and LY341495 on dopamine transmission were AMPA-dependent effects. Other studies have suggested that the effects of ketamine on dopamine transmission cannot be attributed either to a direct ketamine action on dopamine reuptake, or to an interaction with dopamine receptors (Can et al., 2016). A further support of the involvement of the NAc in the effects of ketamine has been provided by Yao et al. (2018), who have demonstrated that a single injections of a low dose of ketamine induced the impairing of long-term potentiation in the NAc; this effect was maintained for 7 days and was not associated with any alteration of basal synaptic transmission mediated by AMPARs and NMDA receptors. These results were somehow anticipated by Réus et al. (2013) who reported that ketamine but also imipramine, decreased histone deacetylation in the NAc; this function, that is important for long-term changes related to stress and antidepressant treatment, has been found increased in the NAc but not in the PFC, hippocampus, and amygdala in maternally deprived adult Wistar rats. A further confirmation of NAc involvement in ketamine’s actions, has been provided by Abdallah et al. (2017), who have shown that in a MDD subgroup of patients displaying an enlarged bilateral NAc volume, ketamine treatment normalized NAc volume in patients who achieved remission.

### Lateral Habenula

The fourth area that we will consider is the LHb, an area that has been implicated in anxiety, stress, pain, avoidance learning, attention, human reward processing, psychosis and depression (Proulx et al., 2014; Gold and Kadriu, 2019). In particular, LHb has been proposed as a source of negative reward-related signals for dopamine neurons (Matsumoto and Hikosaka, 2007), on the basis of recorded neuronal activity in rhesus monkeys during visually guided saccade task, and of the strong inhibition of dopamine neurons after weak electrical stimulation of the LHb. The crucial role of LHb is further strengthened by the observation that acute stress can transform LHb reward responses into punishment-like neuronal signs (Shabel et al., 2019). Based on this evidence, it is likely that mood improvement could be produced through the inactivation of this so-called “anti-reward center,” and therefore it is possible that ketamine could produce its rapid antidepressant actions through modulating LHb activity. In this regard, Yang Y. et al. (2018) reported that the blockade of NMDA receptor-dependent burst activity in the LHb, operated by ketamine, could mediate its rapid antidepressant actions in rat and mouse models of depression, by disinhibiting a downstream monoaminergic reward center. These authors also showed that burst-evoking photo-stimulation of LHb drive despair and anhedonia. Concordant results, obtained in late adolescent male rats, have shown that early life stressors such as maternal deprivation, can produce LHb intrinsic excitability and LHb bursting activity, that were associated with increased immobility in the forced swimming test; in this model, ketamine was able to persistently reverse maternal deprivation-induced changes in LHb neuronal excitability and firing patterns up to 72 h post injection (Shepard et al., 2018).

Summarizing the above, it emerges that the LHb could play a relevant role either in the depression or in the effects of ketamine. In particular, with its peculiar intrinsic activity and connections with the other brain areas involved in depression, the LHb has been proposed as the site through which the rapid action of ketamine occurs (Cui et al., 2019).

### Subgenual Cingulate Region

The subgenual cingulate cortex (sgACC) also known as subgenual cingulate region or Broadman area 25 (Cg25) is one of the most implicated regions in MDD; it has an important role in emotion regulation, cognition, reward anticipation and anhedonia (Stevens et al., 2011; Rudebeck et al., 2014; Alexander et al., 2019). This area corresponds to the infralimbic cortex in rodent models, although a precise functional homology cannot be demonstrated (Sousa, 2016). Several fundamental information on the brain areas involved in the effects of ketamine can be obtained by evaluating PET (positron emission tomography) or functional magnetic resonance brain imaging of TRD patients undergoing ketamine therapy; correlation between the activation of specific areas and the therapeutic improvements of the patients examined, may allow to include or exclude specific brain areas. Similarly, by examining the therapeutic outcomes or the behavioral effects observed in TRD patients undergoing deep brain stimulation (DBS) it is possible to associate the activation of highly specific brain regions with the magnitude of the clinical improvement (Drobisz and Damborská, 2019). Consequently, the combination of the information obtained with these two approaches can support the role of a specific brain area in depression and allow to understand more deeply the role of specific areas in the antidepressant effects of ketamine. The application of DBS to the Cg25 of TRD patients produced a reduction in the metabolic hyperactivity in this area (observed by PET) and an evident and sustained clinical outcome; these results were described in a seminal report (Mayberg et al., 2005). Similarly, clinical improvement following pharmacotherapy and psychotherapy did correlate with a decrease in Cg25 metabolic activity and interestingly, transient sadness increases Cg25 metabolic activity (Mayberg et al., 1999; Auer et al., 2000; Mirza et al., 2004; Seminowicz et al., 2004). Cg25 has very robust connections with many brain areas implicated in normal and abnormal emotion processing and memory such as NAc, hippocampus, amygdala, hypothalamus, and orbitofrontal cortex (Lozano et al., 2008; Zeredo et al., 2019). Notably, the DBS that was applied to the white matter tracts adjacent to the subgenual cingulate gyrus, induced metabolic changes in various regions; in particular PFC activation and orbitofrontal cortex inhibition were concomitant with a striking and sustained remission of depression in four of six patients at 6 months after DBS (Mayberg et al., 2005; Johansen-Berg et al., 2008). In addition, the SgACC/Cg25 could be a target for ketamine, because of the relevant role of Cg25 in regulating the glutamatergic hypofunction of hippocampus in high trait anxiety (Zeredo et al., 2019) and the ability of ketamine of modulating sgACC connectivity (Wong et al., 2016). In particular the combination of intracerebral microinfusion with cardiovascular and behavioral monitoring in marmoset monkeys showed that overactivation of sgACC blunts appetitive anticipatory, but not consummatory arousal (Alexander et al., 2019); interestingly these authors showed that ketamine treatment ameliorates the blunted anticipatory arousal and reversed the associated metabolic changes in sgACC. Interestingly, S and R ketamine induced metabolic changes in the brain of healthy volunteers receiving a sub-anesthetic intravenous dose (1.0 mg/Kg) (Vollenweider et al., 1997). In particular, S-ketamine treatment increased glucose cerebral metabolic rate (CMRglu) in cortical brain regions 2–3 times more than in subcortical regions (Vollenweider et al., 1997); likewise, frontal regions were stimulated about twice as much as posterior regions (Vollenweider et al., 1997).

The score for psychiatric alterations such as hallucinatory-disintegration, ego-dissolution and mood changes did positively correlate with CMRglu in the occipital cortex, cingulate cortex, frontomedial, temporomedial and frontolateral cortex. A recent study, based on glucose metabolism assessment (^18^F-FDG/PET) in TRD patients, 40 min after intravenous injection of ketamine at 0.2 and 0.5 mg/Kg, reported a significant increase in the metabolic activity of the anterior cingulate area, posterior central gyrus, supplementary motor area and prefrontal cortex (Li et al., 2016). Therefore, based on this evidence, it may be concluded that S-ketamine amelioration of depressive symptoms, may be highly complex as it involves many areas when compared to the equally effective localized activation of the dorsolateral prefrontal cortex by DBS. It may be worth noting that a similar pattern of metabolic stimulation has been observed with psilocybin, a drug that has been recently used in TRD patients (Carhart-Harris et al., 2017). The metabolic and psychotic effect of ketamine and psilocybin has been suggested to be due to their action on a common pathway processing sensory and cognitive information (Vollenweider et al., 1997). A comparison of the neuropsychological effects in healthy subjects and in patients with MDD or TRD and the study of the therapeutic response in the latter, observed after administration of (R,S)-, (R)-, or (R)-ketamine, might help to understand the importance of ketamine-activated brain areas that are not relevant to remission of depression. As suggested above (see sections “ESKETAMINE: CLINICAL USE IN DEPRESSION” and “KETAMINE AND SUICIDAL BEHAVIOUR”), the effect of ketamine may differ depending on the mood state of the individual. On this basis, we can speculate that the rapid response of ketamine in responsive patients suggests that it is capable of activating brain circuits that may be only temporarily deactivated, and that this type of response may not be observed when ketamine is taken by healthy volunteers; reversibly circuits that are activated in the latter may not be available in MDD or TRD individuals. Interestingly, different enantiomers of ketamine might produce different effects and these effects might interact when (R,S)-ketamine is administered as reported by Passie et al. (2021) who suggested that (S)-ketamine and (R,S)-ketamine differ somewhat regarding their psychopathological effects. This observation may be relevant when evaluating the effects of ketamine in patients with TRD.

Deep brain stimulation of other brain areas such as the NAc, has produced immediate and unprompted clinical improvement in major depression with no adverse effects (Schlaepfer et al., 2008); metabolic changes similar to those observed during stimulation of Cg25 were observed following 1 week of stimulation. This effect was not surprising because supported by the connection between the Cg25 and the NAc and those between NAc and other brain areas (Schlaepfer et al., 2008); in particular in the amygdala and in the NAc itself, the metabolism was also increased. The improvement of the clinical ratings in TRD following stimulation of the NAc may help understanding how ketamine may modulate brain activity, since it is unclear how the inhibition of NMDA receptors function may produce a widespread increase of CMRglu. However, it has been suggested that the block of NMDA receptor on inhibitory interneurons by S-ketamine, may facilitate pyramidal cells firing leading to CMRglu cortical increase (Hashimoto, 2019) more elements must be considered. The gabaergic medium spiny neurons of the NAc receive inputs from dopaminergic neurons located in the VTA and from glutamatergic neurons originating in the amygdala, the hippocampus the paraventricular nucleus of thalamus and the medial PFC (Pinto et al., 2003; Pierce and Kumaresan, 2006). In turn, the NAc projects indirectly to several regions including Cg25, ventral pallidum, thalamus, hypothalamus, and feeds back on the amygdala and the medial PFC (Schlaepfer et al., 2008), generating a sophisticated circuitry where the unbalance of one component may unsettle many other brain regions.

The amelioration of depression by either DBS or ketamine in TRD patients shares the activation of peculiar brain areas, whereas it is conceivable that synaptic long-term depression may be more abundant than in healthy subjects. On the other hand, brain areas of TRD subjects have been found to be hyperactive (e.g., Cg25) and their activity resulted decreased upon therapeutic intervention. At this regard, Morris et al. (2020), through an elegant fMRI study, have shown that MDD patients had higher sgACC activation to positive and negative monetary incentives compared with controls, and that ketamine reduces sgACC hyper-activation to positive incentives (associated with anhedonia) but not negative incentives (associated with anxiety). Furthermore, in TRD subjects, an open-label PET study performed before and 2 h after ketamine infusion reported a reduction of anhedonia correlated with increased glucose metabolism in the hippocampus and dorsal anterior cingulate cortex (dACC) and decreased metabolism in the inferior frontal gyrus and orbitofrontal cortex (OFC) (Lally et al., 2015). It may possible that in hyper active brain areas, the overactive NMDA receptors might be the target of low therapeutic doses of ketamine because their affinity for ketamine might be higher (Shaffer et al., 2019). While little differences in the affinity for ketamine were reported for the four subtypes of NMDA receptors (Yamakura et al., 2000), it is also possible that in TRD subjects, the receptors undergoing turnover may acquire higher affinity for S-ketamine as a consequence of an editing process (Barbon and Magri, 2020). It has been reported that the turnover of AMPA receptors may be stimulated by ketamine and by its metabolites (2R,6R)-HNK and (2S,6S)-HNK (Zanos et al., 2016; Ho et al., 2018). Editing at this receptor is well known to occur to provide calcium permeability (Wright and Vissel, 2012) and it would be interesting to know whether the activation of AMPA receptors is a phenomenon that occurs preferentially on the known edited version or on a different version that occurs in TRD patients before or after ketamine exposure.

## Ketamine and HPA Axis

It is well known that depression is generated by the combination of genetic and environmental factors; among these, chronic stress has a pivotal role in humans and in animal models of depression (Czéh et al., 2016). Although the consequences of chronic stress on parameters such as hippocampal neurogenesis, BDNF levels, monoamine transmission and neuroinflammation are well known (Kubera et al., 2011; Kim and Won, 2017), it is somewhat less clear the relationship between the above mentioned parameters and the dysfunction of the HPA axis, and the correlated increase of cortisol, generated by chronic stress (Juruena et al., 2018).

In general it is known that chronic stress is closely related with depression (Hammen, 2005). One wonders how the activation of the HPA system and changes in cortisol levels and glucocorticoid receptors in many brain areas are related to the manifestation of depression (Pariante and Miller, 2001; Farrell and O’Keane, 2016; McEwen and Akil, 2020; Rothe et al., 2020) and what the role of mineralcorticoids might be (de Kloet et al., 2016). Interestingly, the individual response to HPA activation differs in the two sexes and especially differs between susceptible and resilient individuals (Kokras et al., 2019; Homberg and Jagiellowicz, 2021). These differences manifest in a different propensity to develop depression, and in particular, the depression that occurs may be differently correlated with cortisol levels or glucocorticoid receptors levels. Patients affected by MDD commonly have the HPA system activity set at a higher point, therefore, both the glucocorticoid receptor signaling and the activity of corticotrophin releasing hormone (CRF) neurons are more elevated than in physiological condition (Ströhle and Holsboer, 2003). In particular, levels of cortisol have been considered for assessing the condition of depression and for predicting the result of the antidepressant therapy. The severity of depressive symptoms is generally correlated with cortisol levels (Zobel et al., 2001), but different subtypes of depression may also be associated with higher baseline cortisol levels (Keller et al., 2006). Interestingly there is a difference in HPA-axis activation between melancholic and atypical depressive subtypes; in particular hypercorticolism is associated with melancholia while normal or decreased HPA-axis function should be primarily associated with atypical depression (Juruena et al., 2018). In should also be noted that the response to antidepressant therapy varies differently and correlates differently with HPA-axis activation (Anacker et al., 2011; Ventura-Juncá et al., 2014; Jain et al., 2019). However, understanding the correlation between the type of depression, and the activation of the HPA system, could allow us to predict individual’s response to antidepressants and help to identify the most appropriate antidepressant to achieve a therapeutic response (Nandam et al., 2020; Nikkheslat et al., 2020). The meaning of cortisol levels in depression and in the response to antidepressant therapy has been recently reviewed elegantly by Nandam et al. (2020); these authors, examining the role of HPA activation in animal models of depression, concluded that there is no convincing relationship between cortisol level and therapeutic response, in either preclinical or clinical setting. As far as regards the correlation between the antidepressant effects of ketamine and changes in cortisol levels, no clinical or experimental results are yet available. However, an interesting work has pointed to Mg^2+^, as a link between ketamine antidepressant actions and cortisol levels (Murck, 2013); this study compared the action of ketamine with that of high doses of Mg^2+^ in animal models of depression, observing that both led to synaptic sprouting and strengthening. In addition, it was observed that neuroendocrine changes (i.e., increased cortisol and aldosterone) were associated with low levels of Mg^2+^ and that patient with therapy refractory depression appeared to have lower CNS Mg^2+^ levels in comparison to healthy controls (Murck, 2013). On the other hand, it is interesting to observe that chronic corticosterone treatment provides a useful animal model of depression, and that multiple classes of antidepressants can reverse the neurogenic effects observed in this model (Levinstein and Samuels, 2014). On this basis, and considering that acute and chronic ketamine can reverse the effects of chronic mild stress (CMS) and the increased levels of circulating corticosterone and ACTH, it is likely that ketamine might have similar effects in humans and in the corticosterone model of depression. Nevertheless, the exact mechanism by which ketamine might correct dysfunctions in the HPA system has not yet been identified.

## Role of BNST in the Antidepressant Effect of Ketamine and Standard Antidepressants

The preclinical studies on the possible use of ketamine in the therapy of depression have been mainly based on animal models of depression that have been validated over the years, through the use of classic antidepressants (Czéh et al., 2016; Planchez et al., 2019). Although the mechanism of action of ketamine (Zanos and Gould, 2018; Deyama and Duman, 2020; McIntyre et al., 2020) differs substantially from that of standard antidepressants (Feighner, 1999; Patel et al., 2017), it might be hypothesized that they share common target areas that therefore could be involved in the etiology of depression. This hypothesis might be considered to explain the antidepressant effect produced by selective serotonin reuptake inhibitor (SSRIs), and by selective norepinephrine reuptake inhibitor (SNRI), which, although acting on two different transmission systems, can produce equivalent antidepressant effects (Artigas, 2015; Gałecki et al., 2018; Wagner et al., 2018). In addition to the brain areas discussed above, the bed nucleus of stria terminalis (BNST) should be considered as a target of antidepressant action. The BNST has been included in the extended amygdala and plays a relevant role in the acquisition of emotions and in motivated behavior (Alheid et al., 1998); in this regard a diminished motivation is an essential feature of depression (Nestler and Carlezon 2006). BNST also plays a role in the integration of stress and reward information (Carboni et al., 2000; Walker et al., 2003; Choi et al., 2008) and in stress-induced relapse of drug seeking (Erb et al., 2001; Sahuque et al., 2006; Jadzic et al., 2021). In addition, BNST is implicated in the regulation of fear, anxiety and aversion (Davis et al., 2010; Kim et al., 2013; Jennings et al., 2013); noteworthy the electrical stimulation of BNST produces both excitatory and inhibitory responses in VTA neurons *in vivo* (Georges and Aston-Jones, 2001). Therefore, based on the strict connection of BNST with brain areas involved in depression and anhedonia, it might be assumed that BNST plays a relevant role in depression etiology, as well as in the mechanism of action of antidepressants. This hypothesis is supported by the observation that patients affected by TRD could benefit from DBS of the BNST (Fitzgerald et al., 2018), although a larger clinical study will be needed to confirm the results of this pilot study. Remarkably, BNST receives a very dense noradrenergic innervation that originates from the A2 region of the nucleus of solitary tract, and the A1 region of the caudal brainstem, with a small contribute from the locus coeruleus (Aston-Jones et al., 1999; Delfs et al., 2000), all these areas being involved in acute and chronic stress response (Forray et al., 1997) and in arousal (Herman et al., 2018). A role of this brain area in depression is also supported by the serotoninergic innervation of the BNST; specifically, it has been reported that the availability of serotonin transporters in this area is positively correlated with individual differences in anxiety behavior (Oler et al., 2009). We have previously observed that classic antidepressants (Cadeddu et al., 2014) and ketamine (Cadeddu et al., 2016) shared the ability to increase catecholamine output (i.e., extracellular concentration) in the BNST; intriguingly, the SSRIs citalopram and fluoxetine increased the output of norepinephrine and dopamine similarly to the selective blockers of norepinephrine reuptake such as desipramine and reboxetine, suggesting a converging pathway of activation of catecholamine transmission in this brain area. On the other hand, a sub-anesthetic dose of ketamine (i.e., 10–40 mg/kg i.p.), dose dependently increased the output of norepinephrine and dopamine up to about 180% of basal values. Remarkably, Tso et al. (2004) observed that S and R ketamine at 100 μM, similarly increased norepinephrine efflux and the t^1/2^ uptake in rat BNST slices although it should be noted that 100 μM is well above the therapeutic concentration of ketamine in humans, and this concentration might interact with other receptors besides NMDA. In general, norepinephrine and dopamine innervations make synaptic contacts on CRF-neurons, which in turn influence glutamate release from afferents on GABA BNST neurons, producing a disinhibition of VTA neurons (Kudo et al., 2012; Silberman and Winder, 2013). In addition, the output neurons of the ventral BNST are under norepinephrine tone (Forray and Gysling, 2004), whose increase by the administration of the α-2 antagonist yohimbine contributes to the activation of HPA (Zheng and Rinaman, 2013). In synopsis, it is likely that BNST plays a role in the antidepressant effects of ketamine. The fact that both ketamine and classic antidepressants increase catecholamine output in the BNST suggests that this effect is necessary for the generation of the antidepressant effect, although it might not be sufficient for the generation of the fast antidepressant effect of ketamine. In addition, the selective effect of ketamine in specific brain regions could also be an important for its rapid antidepressant properties.

## Ketamine’s Effects: Gender and Development

When discussing about depression and antidepressants, the attention is focused on the relative low response of depressed patients, and the lag-time in the response. In addition, some attention has been given to the higher incidence of depression in women as compared with men, and to the different responses of women to the antidepressant therapy. We will consider here the background underlying women’s different response to ketamine. MDD, assessed by DSM-IV criteria has a lifetime prevalence of 11%, or 7.5% when referred to the past year (Avenevoli et al., 2015). Depression has a higher prevalence (2:1) in women (Kuehner, 2017), both when assessed in adulthood and in adolescence; in particular, non-severe and severe MDD in adolescents reached 12% and 5% in girls and 7% and 2% in boys, respectively (Avenevoli et al., 2015). Although social and cultural factors could have a role in generating these differences, several studies have attempted to elucidate the neurobiological bases potentially implicated. In addition to being affected by depression to a greater extent, women attempt or complete suicide with higher incidence (Mergl et al., 2015). Whether the origin of this difference is genetic, environmental, or is due to the interaction of these two factors is an important open question. Moreover these two factors may be additive; in fact, women have a higher sensitivity to stress (Slavich and Sacher, 2019) but are also more frequently victims of stressful situations such as domestic violence, unsatisfactory employment conditions and home keeping burden (Beydoun et al., 2012). Looking at the neurochemical bases of women’s different susceptibility to depression, we can underscore differences in the brain systems involved in stress handling, in the release of CRF, and in general, in the norepinephrine circuitry, as compared with men (Bangasser et al., 2016; Bangasser and Wiersielis, 2018). While the acute stress response can be considered an adaptive response, and usually does not lead to any hampering dysfunction, persistent activation of stress circuits can lead to a pathologic hyper-arousal which can evolve in a stress-related psychiatric disorder (Gold, 2015). In particular, the locus coeruleus neurons in rat respond with greater activation to stressors when they have been previously sensitized by CRF release during stress; remarkably in non-stressed situations, there is no tonic CRF release (Curtis et al., 2006).

The different gender sensitivity that is observed in the prevalence of depression also extends to the response to antidepressant therapy. In fact, women respond differently to antidepressants, and in particular, they respond better to SSRI than men, while men respond better to tricyclic antidepressants (LeGates et al., 2019). Such a response of women to SSRI is not seen in old women, who improve their response when a hormonal replacement therapy is administered (Thase et al., 2005). At present there is no clear evidence of a gender difference with regard to fast acting antidepressant therapy because it is use is currently limited, but based on animal studies, one should expect a better response in women (Freeman et al., 2019). In the forced swimming test, naïve females show an antidepressant response (Carrier and Kabbaj, 2013) to a lower ketamine dose than males; this response was shown to be estrogen- and progesterone- dependent. Remarkably, these authors found that the higher sensitivity of female to low doses of ketamine was not mediated by the phosphorylation of mTOR in the medial prefrontal cortex, or by the eEF2 in the hippocampus. Additionally, Ardalan et al. (2020) observed that (S)-ketamine, one hour after administration (15 mg/kg i.p.), induced a substantial alteration of morphology of the hippocampal astrocytes only in females of a genetic animal model of depression, the Flinders Sensitive Line rats. In this regard, it is interesting to note that, in female mice, both estrogen and progesterone increase spine density in the hippocampus, a brain area that expresses high levels of receptors for these hormones (Li et al., 2004). Furthermore, Thelen et al. (2019) observed that the repeated administration of ketamine (10 mg/kg, 21 days) to C57BL/6 mice, produced an increase in the synaptogenic response in the hippocampus of female mice, while ketamine induced a sex-specific “glutamate burst” in the male medial PFC. On this basis, it can be summarized that women have a higher susceptibility to stress and therefore are exposed to a higher grade of anxiety and other depressed mood-related disorders, because their brain structure is under the influence of hormonal fluctuation during the reproductive years, and in the midst of hormonal transition period that occurs later in life. At the same time, women are at great risk for developing inflammatory-related depressed mood because the sex differences in the human immune response (Slavich and Sacher, 2019). Therefore, it is not surprising that women can respond differently to ketamine therapy.

## Ketamine and Inflammation

There is strong evidence that inflammation is implicated in the pathophysiology of depression, and although the heterogeneity of the results on its role, it appears that managing inflammation might provide an overall therapeutic benefit, regardless its origin (Beurel et al., 2020). A recent meta-analysis study revealed that several cytokines such as IL-6, tumor necrosis factor (TNF)-α, IL-10 were elevated in MDD patients, while Interferon-γ was reduced (Köhler et al., 2017). The inflammatory markers such as IL-6 and CRP/hsCRP (C reactive protein/high sensitivity CRP) have been suggested as markers for the prediction of treatment-response in TRD patients (Yang et al., 2019b). A second component of central inflammation is the activation of brain microglia cells which, through the release of IL-1 and TNF-α, IL-1β and other inflammatory mediators, can directly modulate the glutamate transmission (Riazi et al., 2015). At the same time, central inflammation may interact with other neurotransmitters and neurocircuits, leading to behavioral changes (i.e., sleep, motivation, reward and anhedonia) and depressive symptoms; therefore, targeting inflammatory mechanisms can offer new therapeutic and diagnostic opportunity (Roman and Irwin, 2020). Noteworthy untreated depressed individuals had elevated plasma and cerebrospinal fluid levels of CRP, elevated levels of glutamate in basal ganglia and showed increased anhedonia and psychomotor retardation (Haroon et al., 2016; Felger et al., 2020). Moreover, lipopolysaccharide mediated inflammation induced depressive-like behavior that was ameliorated by the administration of low doses of ketamine (Walker et al., 2013). In another study it was shown that a single dose of ketamine restored the lipopolysaccharide-induced depressive-like behavior (increased anxiety and reduced self-care), reduced the cytokine production, the microglial activation and the microglial quinolinic acid production (Verdonk et al., 2019). These authors also showed that in TRD patients, the kynurenic to quinolinic acid ratio is a predictor of ketamine response, and the reduction of quinolinic acid after ketamine infusion, is a predictor of the reduction of MADRS (Montgomery-Asberg Depression Rating Scale) score, thus supporting the hypothesis that microglia is involved in the pathophysiology of depression (Verdonk et al., 2019). A further support of this hypothesis has been provided by Ho et al. (2019), who have shown that in HMC3 human microglial cells, either ketamine or its active metabolites (2R,6R;2S,6S)-HNR, can regulate type I interferon pathway. Furthermore, these authors suggested that signal transducer and activation of transcription factor 3 (STAT3) may play a role in the antidepressant effects of ketamine, mediating the increase of BDNF expression and promoting the synthesis of synaptic proteins such as PSD95 and synapsin-1 (Ho et al., 2019).

The involvement of inflammation in the etiopathogenesis of depression has aroused considerable interest and has extended the search for possible interactions between other conditions that are generally associated with inflammation and depression, such as obesity and type 2 diabetes. Wang F. et al. (2019) examined this issue in a systematic review, concluding that the prevalence of major depressive disorder in people with type 2 diabetes was elevated and significantly higher than that in the general population. A further factor that correlates obesity with inflammation and depression is the reduced physical activity, which is a common denominator of obesity, metabolic syndrome and depression (Schmidt et al., 2020). In particular, Dutheil et al. (2016) observed that an increase in inflammatory cytokines (IL-6, IL-1β, TNF-α) was associated with an increase in anxiety and anhedonia symptoms in mice subjected to a high-calorie, high-fat diet; these authors also highlighted the mechanisms involved, showing that the behavioral changes observed were associated with a disrupted insulin signaling in the hippocampus, combined with elevated serum corticosterone. In addition, they showed that high-fat diet caused an altered energy homeostasis and an altered insulin/mTORC1-signaling pathway, that is involved in synaptic plasticity; interestingly, they also observed that ketamine rapidly reversed the behavioral deficits that were caused by long-term exposure to high-fat diet (Dutheil et al., 2016). Curiously, the relationship between depression and inflammation is most evident in TRD patients (Haroon et al., 2018); in particular these authors found a significant relationship between number of failed treatment-trials and inflammation markers such as TNF-α, soluble TNF receptor 2 and IL-6. Considering that these patients are those who show a better therapeutic response to ketamine, it is interesting to evaluate whether the action of ketamine occurs through neuronal mechanisms or directly through the modulation of inflammatory processes (Roman and Irwin, 2020).

In general, antidepressant treatment significantly decrease peripheral levels of IL-6, TNF-α and IL-10, but this reduction, as evaluated by a meta-analysis, is not associated with treatment response (Köhler et al., 2018). Remarkably, elevated levels of circulating markers of inflammation predict a positive response to tricyclic antidepressants, ketamine and electroconvulsive therapy and a poor response to selective SSRI (O’Brien et al., 2007; Yoshimura et al., 2009; Carvalho et al., 2013); in particular, patients with SSRI resistant depression had significantly higher production of the pro-inflammatory cytokines IL-6, TNFα, and IL-6R compared with healthy controls. On the other hand, ketamine administration in TRD patients can reduce plasma levels of the pro-inflammatory adipokines and resistin, supporting the view that ketamine’s anti-inflammatory effects may directly contribute to its rapid antidepressant effects (Machado-Vieira et al., 2017). Moreover, in this study it was observed that low levels of the anti-inflammatory and insulin sensitivity promoter adiponectin, significantly predicted ketamine’s antidepressant efficacy. Overall it can be stated that TRD patients have elevated plasmatic levels of inflammatory cytokines such as IL-6, and this may predict a positive response to ketamine treatment. However, although ketamine does lower IL-6, such a reduction does not always correlate with the antidepressant response (Kiraly et al., 2017), suggesting that further studies are needed to fully elucidate the interaction between ketamine, inflammation and the antidepressant response.

## Ketamine’s Effects: Single Versus Repeated Administration, General Side Effects, Cognitive Effects and Potential Addictive Effects

Several studies have shown the effectiveness of a single administration of ketamine (usually 0.5 mg/kg over 40 min infusion) in reducing, either depressive symptoms or suicidal thoughts for a review see De Berardis et al., 2018; Phillips et al., 2020). Although this evidence has an enormous potential of innovation in the therapy of depression, the real manageability of this potential can be fully verified only through further studies and clinical trials. Furthermore, these studies have shown that the antidepressant effect of ketamine, can be assessed up to 10 days from the single administration but how depression symptoms evolve months or years later, in terms of intensity and frequency remain largely to be ascertained because this was not among the objectives of these studies. In general, because a single dose ketamine is short lived, 50% of patients relapse within one week, although 20% remain in remission up to 30 days (Salloum et al., 2020). Therefore, it is pivotal to evaluate the effect of repeated ketamine treatment with the final goal of developing a chronic treatment that does not produce addictive or psychotropic effects, or in general, significant side effects. The most common dose of i.v. ketamine is 0.5 mg/kg (0.25 for S-ketamine) over 40 min but some patients respond to doses as low as 0.1, while others may require up to 0.75 mg/kg (Andrade, 2017). The recommended intranasal dose of esketamine is 28 mg/device; each device delivers 2 sprays for a total 28 mg, to be administered twice per week; the bioavailability has been reported to be about 45% (Yanagihara et al., 2003) or almost complete (Andrade, 2017). The bioavailability of oral ketamine is low (8% for esketamine and 24% for ketamine) thus a dose of about 2.0-2.5/kg is equivalent to the i.v. 0.5 mg/kg dose (Andrade, 2019). A thorough description of the pharmacokinetics of ketamine is beyond the scope of this review and we refer to excellent work of other authors (Yanagihara et al., 2003; Fanta et al., 2015). The peak plasma concentration (min) of the two ketamine enantiomers (a) or of the two nor-metabolites (b) was detected and reported as a:b) in min is about: 0:30, 30:90, 20:80, 30:60, 20:30 after injection, intranasal administration, sublingual tablets, tablets and suppository, respectively (Yanagihara et al., 2003). An early study (Shiroma et al., 2014) reported that the repeated administration of ketamine over a period of 12 days (i.v. infusion of 0.5 mg/kg over 40 min, thrice a week) determined 100% of response and about 60% of remission; in addition, it was shown that 50% of patients relapse (i.e., improvement less than 50% in baseline, as evaluated by the MADRS score at visit). Another study (Vande Voort et al., 2016), although of small size (12 subjects), has shown that 7 subjects (58.3%) responded to ketamine treatment during the acute phase (0.5 mg/kg over 100 min i.v. infusion) and 5 subjects (41.7%) remitted; these 5 subjects were then administered with further 4 week ketamine infusions (thrice a week) and experienced further depressive symptom improving, during the continuation phase treatment, as evaluated through MADRS score. Interestingly, Vande Voort et al. (2016) reported that four subjects lost remission status during the drug free post-continuation phase, but they maintained a MADRS total score not different from that evaluated 24 h after the first acute infusion suggesting an enduring ketamine effect. A recent meta-analysis of 20 randomized and controlled studies evaluated the efficacy of a single or repeated ketamine dose in different subgroups of patients with MDD and bipolar depression (Kryst et al., 2020); the authors reported that a single dose of ketamine, reduced depressive symptoms, producing the largest antidepressant effect at 24 h, however, a significant effect was seen up to 7 days after ketamine administration. In addition, ketamine’s effect could be observed in TRD patients, who received ketamine in monotherapy, but also when ketamine was used as adjunctive to the current antidepressant therapy, in both unipolar and bipolar depression. Several studies evaluated the efficacy of ketamine repeated treatments; importantly, serial ketamine administration (twice or thrice a week for three weeks) produced a significant and sustained antidepressant effect over placebo at three weeks, both in terms of depression symptoms and in terms of remission (Kryst et al., 2020).

On the basis of this evidence it is possible to predict that the repeated administration of ketamine represents a concrete therapeutic response to depression and suicidal ideation. However, it remains to be evaluated whether the oral or intranasal route of administration could be a valid alternative to the intravenous one; moreover, the consequences of long-term exposure to ketamine remain to be monitored. The efficacy and safety of esketamine nasal spray was evaluated when added to a newly initiated oral antidepressant in TRD patients, in a randomized double-blind active controlled study (Popova et al., 2019); the authors observed a clinically meaningful improvement in the esketamine plus antidepressant group, while the most common side effects (dissociation, nausea, dysgeusia and dizziness) were observed shortly but were resolved by 1.5 h after dosing. Similar results have been reported by Daly et al. (2019) in a clinical trial, involving 297 randomized TRD patients, aimed at assessing relapse prevention; the authors observed that, among patients administered with 56-84 mg of esketamine nasal spray plus an oral antidepressant, 80% of patients who achieved stable response were without relapse 11 weeks after the first administration (versus 58% in the oral antidepressant/placebo nasal spray group), whereas after 70 weeks the percentages were 65 and 35 respectively. Finally, a very recent study (Ionescu et al., 2021) evaluated the efficacy of esketamine nasal spray [84 mg or placebo + standard of care (SC)], administered twice a week for four weeks; at day 25 the authors observed a 47% of remission, in patients who received ketamine + SC, versus 37% in the placebo + SC group. In addition, the authors observed that the most frequently reported adverse effects, in ketamine + SC were: dizziness (41.2%), dissociation (38,6%), nausea (33.3%), dysgeusia (25.4%), somnolence (22.8%), and headache (21.9%); interestingly the majority of adverse effects in the esketamine + SC group (89%) and placebo + SC group (68%) were reported on intranasal dosing days and most of these events (94.9% and 84.9%), resolved on the same day they began (Ionescu et al., 2021). A recent study has evaluated the oral administration route of esketamine, as add-on to regular antidepressant medication, in a randomized controlled trial (Smith-Apeldoorn et al., 2019); oral esketamine administration to TRD patients (10 to 30 mg, three times a day over 40 days) was effective and well tolerated. In this regard it should be remarked that the absorption of oral ketamine appears to vary substantially both between and within patients and that ketamine undergoes extensive first-pass metabolism resulting in low and variable bioavailability if compared with IV administration route (Smith-Apeldoorn et al., 2019). A meta-analysis of studies that evaluated the use of oral ketamine for bipolar and unipolar depression (Rosenblat et al., 2019), observed that ketamine administration has significant antidepressant effect with good overall tolerability although antidepressant effects were not as rapid as those associated with IV ketamine; in addition, the author concluded that a reduction of suicidal behavior and efficacy in TRD patients have yet to be demonstrated. On this basis, it appears that ketamine oral administration could represent the easiest and less expensive route of administration, but the limited number of studies does not allow predicting whether the problems associated with this route of administration will be overcome. In particular, the problems of pharmacokinetic and the development of a formulation that can prevent abuse are difficult challenges; in fact, the brain concentration of ketamine needed to achieve the antidepressant effects at the moment it is not known. Overall it can be acknowledged that the repeated administration of esketamine nasal spray in addition to an oral antidepressant is certainly effective and safe, while the side effects are acceptable and short-lived.

Among the CNS adverse effects of ketamine, the most disturbing are the risk of abuse and the effects on cognitions. This issue is stimulating because it is acknowledged that depression is frequently associated with impairments in cognition (i.e., memory and learning) and executive functions (i.e., planning, decision making and mental flexibility) see Culpepper et al. (2017) for a review. Interestingly, TRD patients with low neurocognitive performance level, benefit from a better antidepressant effect of ketamine, and do not exhibit cognitive impairments whereas, TRD patients with elevated cognitive performance, were more likely to show cognitive deficits in working memory and processing speed (Murrough et al., 2015). Furthermore, very recently Shiroma et al. (2020) reported that most, although not all, short-term neurocognitive functions remained stable or improved after repeated (six infusions) or single ketamine administration; in particular, the authors found that there was a greater differential effect of treatment on speed of processing, set shifting and spatial working memory that favors subjects in the six ketamine infusion group (Shiroma et al., 2020). The issue of the safety of repeated ketamine administration has been elegantly reviewed by Strong and Kabbaj (2018); interestingly, the authors suggested that men are more sensitive to the psychomimetic effects of ketamine than women while adolescent and adult female subjects may be more sensitive to the addictive effects of ketamine.

## Ketamine and Depression Therapy: Cure or Treatment?

An essential consideration in the therapy of depression is understanding whether treatment with antidepressants only induces the regression of symptoms or whether it can activate a process of brain neuronal rewiring which, when completed, can lead to a proper healing of the illness. In this process there are two important factors to take into account: (a) the level of influence of the genetic component; (b) the influence of environmental factors. If the effect of the genetic component is sufficient to cause a state of depression, as in fact can occur in late adolescence in absence of other apparent causative factors, it is unlikely that a period of therapy would be sufficient to ameliorate or eradicate symptoms of depression; and even if this were to happen, it is hard to predict whether or not there would be a relapse after the suspension of therapy. Therefore, in cases where there is a lack of response to standard antidepressant therapy and following careful evaluation of a patient’s history, ketamine therapy can be considered as an add-on drug, although a careful assessment of the possible consequences of long-term treatment administration must be carried out.

On the other hand, we know that although insufficient to trigger symptoms, the genetic component plays a significant role in causing depression; in such cases, the tipping point to reaching the threshold for the onset of depression symptoms could be induced by a trauma or by a moderate but chronic stress. Thus, it is possible that the condition of TRD manifests as a result of ineffective standard antidepressant therapy for the removal of traumatic memories or by the persistence of the stressful environmental conditions that have triggered depression symptoms. The use of ketamine as an add-on drug can help to erase memories of traumas and thus eliminate or attenuate nightmares, reduce the insomnia associated with the occurrence of nightmares (Wang X. et al., 2019), and assist recovery from depression. It has in fact been observed that ketamine infusion can increase total sleep time and reduce waking during the first and second night post infusion (Duncan and Zarate, 2013); ketamine can also reduce nocturnal sleeplessness in depressed patients with suicidal tendencies (Vande Voort et al., 2017). Interestingly, it has also been observed that baseline insomnia can be a predictor of the efficacy of ketamine when it is repeatedly administered intravenously for the treatment of unipolar and bipolar depression (Liu et al., 2020). Additionally, repeated ketamine infusions, in a comorbid population with PTSD and TRD, have proven to be an effective treatment (Albott et al., 2018). Overall, although repeated administration of ketamine is very promising for the treatment of PTSD, additional studies are needed to evaluate whether ketamine might enhance the efficacy of psychotherapy in individuals with chronic PTSD (Feder et al., 2020).

On the other hand, although ketamine can produce significant improvements in alleviating chronic stress-associated depression, there is little guarantee that the curative effect will persist once ketamine administration is suspended. In fact, if conditions such as financial hardship, job loss, and/or permanent family or health problems do not find a solution, it is unlikely that depression will miraculously vanish and make any further AD administration unnecessary. There is a shared belief that the therapeutic effects of antidepressants are mediated by the stimulation of neurotrophic factors and synaptogenesis in several brain areas, and can counteract the stress-induced synaptic loss that is considered a root cause of depression (Serafini et al., 2014). In the light of the above, it does seem a concrete prospect that depressed patients will find repeated treatment with ketamine beneficial, even though the cause that generated the chronic stress is still present; nevertheless, it should be taken into account that the administration of ketamine may be necessary until the cause of stress is eliminated, or the environmental situation responsible for depression has significantly improved (Deyama and Duman, 2020). It has in fact been observed that ketamine can induce significant stress resilience in several mice models of depression (i.e., chronic stress, social defeat, learned helplessness) and chronic corticosterone) (Brachman et al., 2016); in view of this, the authors suggested that ketamine may be useful in protecting against stress-induced disorders. In addition, ketamine can generate rapid restoration of synaptic homeostasis, through re-equilibration of glutamate/GABA release and dendritic BDNF mediated reversal of synaptic and brain circuit impairments in stress vulnerable rats (Tornese et al., 2019). Overall, there are tangible possibilities that ketamine or new drugs developed on the basis of ketamine’s mechanism of action, could represent a cure for PTSD and stress related depression disorders, although further clinical data is necessary. This topic has been discussed in a recent review by Abdallah and Krystal (2020), who presented evidence of a synaptic-based chronic stress pathology in depression and other psychiatric disorders.

## Conclusion and Future Directions

This review has shown that ketamine can make a genuine leap forward in the therapy of depression. Its clear effectiveness in reducing symptoms of depression and suicidal ideation, either after a single administration, or especially when administered repeatedly in addition to another antidepressant, is an extremely promising factor in the treatment of depression (Wilkinson et al., 2018; McIntyre et al., 2020). Furthermore, research on new molecules designed to reproduce the rapid and sustained antidepressant effects of ketamine, without its adverse effects, allows us to assume that a new era in the pharmacology of antidepressants has already begun (Chaki, 2017). From a pharmacological point of view, the rapid antidepressant effect and the sustained antidepressant effect of ketamine are both intriguing and puzzling; the former because unlike other antidepressant drugs, it manifests within a matter of hours of infusion; the latter because it continues to be observed well beyond the point when the concentration of ketamine in the plasma is pharmacologically irrelevant. A sustained antidepressant effect has important clinical relevance and has made it possible to schedule repeated treatment at relatively long intervals (48–72 h), which in addition to avoiding any accumulation of the substance, is associated with the appearance of adverse effects only for a short period of time (about 1–2 h) after administration. Existing research on the rapid and sustained action of ketamine has made it possible to broaden our knowledge of the brain areas and circuits involved in the etiopathology of depression and in mood control. Furthermore, these studies have shed light on the role that the lateral habenula plays in the limbic brain circuit that controls motivated behavior (Yang Y. et al., 2018) and on the influence of stress on this circuit. On the other hand, FDA approval of esketamine use in the treatment of depression will probably soon make it possible to ascertain whether ketamine can actually be a cure for depression, at least in a significant number of patients, and at the same time, will allow us to illustrate the risk of its abuse. Finally, the different response of women to ketamine may enable us to better understand the role of hormonal fluctuations in mood control and why depression is highly prevalent in women. On the whole, the repurposing of ketamine in depression therapy and the extensive literature on ketamine’s actions will soon allow not only the identification of new and effective molecules that can be used in depression therapy, but also a greater understanding of mood control, motivated behavior and the role of stress in the expression of these fundamental physiological functions in human beings.

## Author Contributions

EzC wrote the full manuscript except sections “Current Use and Potential for Ketamine Use in Resistant Depression in Adolescence and Childhood,” “Pharmacokinetic Considerations,” and “Subgenual Cingulate Region” and revised the full manuscript. AC contributed to the development and the revision of the full manuscript. ElC wrote the section “Current Use and Potential for Ketamine Use in Resistant Depression in Adolescence and Childhood.” AN wrote the section “Pharmacokinetic Considerations” and “Subgenual Cingulate Region.” All authors approved the final manuscript and agreed to be accountable for the content of the work.

## Conflict of Interest

The authors declare that the research was conducted in the absence of any commercial or financial relationships that could be construed as a potential conflict of interest.

## Abbreviations

AMPA: α-amino -3-hydroxy-5-methyl-4-isoxazolepropionic acid
BDNF: Brain Derived Neurotrophic Factor
BNST: Bed Nucleus of Stria Terminalis
CBT: Cognitive Behavior Therapy
Cg25: Subgenual cingulate region
CMRglu: glucose cerebral metabolic rate
CRF: corticotrophin releasing hormone
CRP: C-reactive protein
DBS: Deep brain stimulation
DHNK: dehydronorketamine
eEF2: eukaryotic elongation factor2
fcMRI: functional connectivity magnetic resonance imaging
HNK: Hydroxynorketamine
HPA: Hypothalamic-pituitary-adrenal axis
LHb: lateral habenula
MADRS: Montgomery-Asberg Depression Rating Scale
MDD: Major depression disorder
mTOR: mammalian target of rapamycin
mTORC1: mammalian target of rapamycin complex-1
NAc: Nucleus Accumbens
NMDA: N-methyl -D-aspartate
PFC: Prefrontal Cortex
PSD95: Postsynaptic density protein 95
PTSD: post-traumatic stress disorder
SC: Standard of care
sgACC: subgenual cingulate cortex
TNF: Tumor necrosis factor
TORDIA: Treatment of Resistant Depression in Adolescents
TRD: Treatment-resistant depression
vHipp: ventral hippocampus
VTA: ventral tegmental area.
